# Serum Therapeutics of Tetanus

**Published:** 1896-09

**Authors:** 


### ABSTRACTS FROM FOREIGN JOURNALS.

UNDER THE DIRECTION OF

J. PRESTON HOSKINS, PRINCETON, N. J.

SERUM THERAPEUTICS OF TETANUS. On the 22d of October Nocard reported to the Académie de Médecine in Paris the first results of his preventive treatment of tetanus. During the first half of the year 1895 he distributed among numerous veterinarians more than 1800 ten-cubic centimetre doses of his anti-tetanus serum, and recommended that in infected districts every animal suffering from a wound (*i. e.*, in danger of getting tetanus) be injected with 10 cubic centimetres of the serum as soon as possible after being wounded, and that the dose be repeated after two weeks. At the time of his report he had received information in regard to 375 animals which had been treated in this way. In all these cases the serum proved absolutely harmless and not one of the inoculated animals suffered from tetanus. All the animals inoculated belonged either to herds in which cases of tetanus had occurred shortly before or stood in the stable with animals afflicted with lockjaw; in several cases the wound had been inflicted at the same time and under the same circumstances as on other patients which were not so treated and which were attacked with tetanus. Finally, the veterinarians who had not lost a single one of the 375 animals treated with serum during the first six months of the year observed during the same period 55 cases of tetanus in animals which were not so treated. In the case of animals already sick with lockjaw treatment with serum is useless.—*Thierärzt. Centralblatt*, May 1, 1896.

